# Evaluation of Emergency First Response’s Competency in Undergraduate College Students: Enhancing Sustainable Medical Education in the Community for Work Occupational Safety

**DOI:** 10.3390/ijerph18157814

**Published:** 2021-07-23

**Authors:** Graciano Dieck-Assad, Omar Israel González Peña, José Manuel Rodríguez-Delgado

**Affiliations:** Tecnologico de Monterrey, School of Engineering and Sciences, Av. Eugenio Garza Sada Sur No. 2501, Col. Tecnologico, Monterrey 64849, Mexico; graciano.dieck.assad@tec.mx (G.D.-A.); jmrd@tec.mx (J.M.R.-D.)

**Keywords:** emergency first aid, educational innovation, higher education, sustainable development goals, learning outside the classroom, safety management, occupational incidents, community medicine, occupational injuries, prehospital management

## Abstract

Worldwide, people’s quality of health has been decreasing due to bad eating habits that have generated an increase in diseases such as diabetes, hypertension, overweight, as well as an increase in hours of the daily workday and stress. This situation can generate sudden illness and work accidents where the need to have knowledge in emergency first response (EFR) is necessary for all. Unfortunately, workshops and courses to certify EFR individuals are usually taught only to healthcare professionals. Therefore, to address this need a EFR project has been developed at the Tecnológico de Monterrey (TEC) which consists of a multidisciplinary challenge to train, certify, and evaluate students’ competency as “emergency first responders” in medical emergencies and healthcare awareness. This EFR project has been performed for one week, every year since 2015, and constitutes a joint venture among academic departments, faculty, and industrial/government institutions, which work together in multidisciplinary projects, providing a source of innovative proposals. The EFR project at TEC has provided instruction and certification for 966 students between 2015 to 2019 and this study has analyzed results considering a sample size of 197 participants. The combination of exam evaluation, medical emergency skills verification, and project proposal results indicate that most students reach skill levels between 2 and 3 in EFR competency after successfully completing the program, regardless of their year of study or the undergraduate program they are enrolled on. This evaluation emphasizes the compromise of the institution and its students in preparation for new living under sanitary conditions for pandemic conditions such as COVID-19.

## 1. Introduction

Studies have been reported to describe improvements for teaching in emergency services in students in medical areas from different approaches. Some examples are: education for mental health in medical services [1]; analysis of teaching in online courses for knowledge transfer [2]; characterizing the knowledge through lay about intervening in symptoms of acute myocardial infarction [3]; evaluating the competencies in emergency management of two groups of medical students, where one of them received training in emergency cases and the other group did not [4]; psychometric evaluations of paramedics as a respective study to analyze the effectiveness in educators [5]; evaluation of the impact of gender on students to measure motivation and learning in students through a digital game teaching content-based emergency first aid [6]; evaluating whether first aid training gives students greater confidence to act in emergencies [7].

Furthermore, the development of evaluations for emergency medical services to professionals has been explored, in order to detect whether the professionals have knowledge regarding how to proceed in cases of incidents where safety knowledge is required, in places where the use of chemistry is developed [8]. Likewise, evaluations have been implemented in short courses of emergency care by technicians in prehospital services [9]; Studies have been carried out documenting the experiences of preceptors who supervise students in their clinical practices in emergency medical services [10]. Similarly, the usefulness of mentoring has been examined in two ways; adaptively based on performance, or based on behavior in smart tutoring for emergency medical services courses [11]. Moreover, comparative studies of teaching methods have been carried out either through a traditional course or workshops about medical emergencies for medical students [12]. Literature review studies have even been reported in international training courses for emergency medical services to synthesize the central elements in order to form a curricular program [13].

With another approach, there is a study to evaluate the content of tweets with the hashtag #FOAMems, since prehospital care physicians and paramedics use this resource as a way of continuing education and for international collaboration [14]. In addition, an analysis of surveys of emergency medical technicians and medical directors has been reported to review the most important basic competencies for the levels of training in medical emergencies in Taiwan [13]. Likewise, a study has been reported to give recommendations on modular training for various educational institutions in the medical area for emergency first aid training [15].

An important area not contemplated in emergency medical services is when it is required to provide massive training for all health professionals; this should be a priority when cases of natural disasters occur, and in that sense a training study of emergency medical services was carried out through lectures to medical students for two weeks [16]. In another study, the implementation of a drill was reported where mass victims were contemplated. In the activity, nursing students, emergency medical technicians, policemen and firefighters were trained in medical emergency services [17]. To maintain certification, first responders must take biennial refresher courses. The certification is granted by the National Registry of Emergency Medical Technicians (NREMT). Such first responder courses are provided by community colleges and/or online education institutions such as Kaplan University, ITT Technical Institute, or Azusa Pacific University.

From the studies, there is an interest in increasing training in emergency medical services for health professionals, where natural disasters that can often be man-made are contemplated. This is due to an increase in demography, which consequently has increased industrialized activity, and the extension of urban areas that cause global warming with this activity. Some examples of these cases can be observed in several earthquakes that have struck Mexico City in 1985 and 2017. Likewise, in 1988, there were tornados in central Florida, or in 2012 and 2017 there were hurricanes with a significantly devastating effect in Louisiana, Florida, and Puerto Rico, respectively. As a result, there are quite a few documented initiatives for creating a community conscience of first responders, even though the evidence has shown that when an emergency, pandemic conditions (such as COVID-19), or disaster strikes, victims and volunteers act as the true first responders. In all cases, ad-hoc citizen groups rescue other people from the rubble and create disaster relief centers, among other emergency response activities.

In addition to this problem, it is in the workplace and in the time invested in transportation that most of the injuries are presented in people. Appendix A of this work presents more details of the global crisis in occupational safety at work worldwide and in Mexico. Therefore, the need to acquire knowledge of EFR is observed regardless of whether people are health professionals. Unfortunately, the available literature related to EFR studies has primarily been focused on training health professionals, and thus ignoring the rest of the society; for instance, among the few studies is one which assesses the attitudes and knowledge of cardio-pulmonary-reanimation (CPR) course to improve prehospital survival rates where high school students aged 15 to 16 years were trained in 18 schools in Hong Kong [18]. Additionally, there is a qualitative study of emergency first aid training in members of communities living in remote areas to generate their confidence and self-efficacy in emergency situations [19]. Another study evaluated the effectiveness of the cabin crew in medical emergency events on flights of an airline in Greece for a period of (2014–2018) [20].

As a matter of fact, a commitment has been made by the countries belonging to the United Nations to work on the 2030 agenda of the sustainable development goals (SDG). For this, universities have begun to adapt their study plans to address the 17 objectives of the sustainable development goals, some examples are described here [21,22,23,24,25]. Among the 17 objectives, objective 3, is “*Health and well-being*”. In particular, objective 3 presents various targets to be met that are presented directly associated with the issue of first aid treatment or medical emergency services that are listed below:

Target 1: Reduce mortality from non-communicable diseases and promote mental health.

Target 2: Reduce road injuries and deaths.

Target 3: Strengthen the capacity of all countries, in particular developing countries, for early warning, risk reduction and management of national and global health risks.

Therefore, it is important to involve all people who are not health professionals so that they can be trained in emergency first response.

As a result, to develop the 17 SDGs, a more proactive initiative is required from the countries, in order to incorporate more knowledge, for instance, in the occupational safety and well-being issue, and all universities must lead the cause. In particular, educators at TEC decided to incorporate this general (common curriculum) competence in all undergraduate programs.

Outcome evaluation has become a very important process in undergraduate competency learning. The Tecnológico de Monterrey (TEC) has taken an important step toward the development of outcome evaluation methods and innovative learning strategies that creates even stronger ties between students and their communities, both nationally and internationally. An example of this teaching process can be observed in the twelfth week of the fall semester (2015 to 2019), in which all undergraduate students from the2nd to the 9th semester of study selected a capstone project activity which allows them to develop high-value activities; as a result, this activity can encourage students in the analysis, implementation and creation of concepts, which would be very difficult to achieve in the classroom.

In addition, the project fosters students’ interactions with real-world organizations and institutions and reinforces specific outcomes that do not necessarily relate directly to their major field of study. In this real-world scenario, students interact with industry professionals, government officials, healthcare specialists, humanists, culture developers, social workers, and others, to propose ideas and procedures to implement solutions to specific problems in their communities. At Monterrey campus, a total of more than 13,000 students participated in this activity called i-Week (in Spanish: Semana-i). i-Week takes place at different places in Mexico and abroad; one activity, of many proposed in i-Week, consists of a training workshop for students to become certified in EFR. Thus, this paper discusses the evaluation of the EFR competency in undergraduate students of all majors that participated in the EFR project (i-Week) from 2015 to 2019. Finally, this study described an unprecedented work in the available literature, which incorporates the development of prototypes or products that students have developed to link their undergraduate programs with the EFR workshop, as part of the evaluation by competencies in the EFR subject; consequently, this is another factor that makes this study unique, as it considers the training, evaluation, and certification in the EFR competency for a population of university students enrolled in all undergraduate areas.

Section 2 shows the structure of the i-Week workshop, the motivation and purpose for this study, the formulation of four research questions, the research quantitative design method, and the sampling methodology used to select the participants contemplated for this study. In Section 3, the quantitative results are presented. In Section 4 a discussion of the study findings is shown, along with the limitations of this study. In Section 5, the conclusions of this study and future work are presented. Finally, in Appendix A and Appendix B, some background information about the workplace occupational safety worldwide and in Mexico is presented, and a description of the inter-institutional administrative structure of EFR i-Week workshop, including its schedule, is briefly discussed.

## 2. Materials and Methods

### 2.1. Structure of the i-Week EFR Workshop

The purpose of this research is to determine the domain level development in EFR competence during the i-Week in undergraduate students from different majors at TEC. Moreover, we evaluate how they can use skills, behaviors, attitudes, and activities to go beyond this, with innovation projects in the field. The EFR outcome includes citizen participation, collaborative work in solving multidisciplinary medical emergency projects, information technology development, and CONAPRA certification. The EFR project promotes active participation [26,27,28,29] with the government health administration to students and faculty members, where they can serve as first responders and even more; and it facilitates training and collaboration using information technologies and mobile apps to disseminate first aid response techniques in medical emergencies. The training consists of a first responder workshop that provides competencies in EFR, basic medical attention, and evaluation of emergency contingencies to aid individuals who have suffered accidents or sudden illness while the paramedics or medical specialists arrive at the scene. Faculty members from computer science, mechatronics, and electrical engineering participated in the project as mentors and instructors during the i-Week. Physical education faculty and healthcare campus representatives were also involved in the process of training and certifications.

The appendix shows the inter-institutional administrative structure and EFR i-Week workshop schedule. Despite the importance of multidisciplinary outcomes in undergraduate education, specifically general education, the evaluation of the impact of these outcomes in undergraduate students’ life education has not been studied before. This study intends to perform a competence evaluation of the multidisciplinary outcome which is not necessarily part of the major undergraduate students’ curriculum, but is very important in his/her lifelong education.

### 2.2. Research Design

This research was guided by the following research questions:

R.Q. 1 What relationship exists between the CONAPRA certification exam performance and the “emergency first response” competence acquisition by undergraduate students?

R.Q. 2 What are student behaviors and attitudes towards the generation of contributions and innovations to the first responder community in Mexico?

R.Q. 3 What differentiation exists between domain levels, 1, 2 or 3, when students are certified by the final grade that includes the CONAPRA exam to obtain the “emergency first response” competence, and their major discipline of study (science/engineering, administrative/management studies, medical professions, or law)?

R.Q. 4 What differentiation exists between domain levels, 1, 2 or 3, when students are certified by the final grade that includes the CONAPRA exam to obtain the EFR competency, and their year of study (freshmen, sophomores, juniors, and seniors)?

For this study, domain level 1 corresponds to the lower level and domain level 3 corresponds to the mature high-performance level. Additionally, student behaviors are defined as decisions that students make regarding genuine interest in understanding first aid skills, developing working exercises, struggling with specific topics, and deciding whether to take advantage of or ignore extra resources available for innovation development during the week. Student attitudes refer to their feelings about citizenship participation in medical emergencies, wherever they may be.

**Figure 1:**
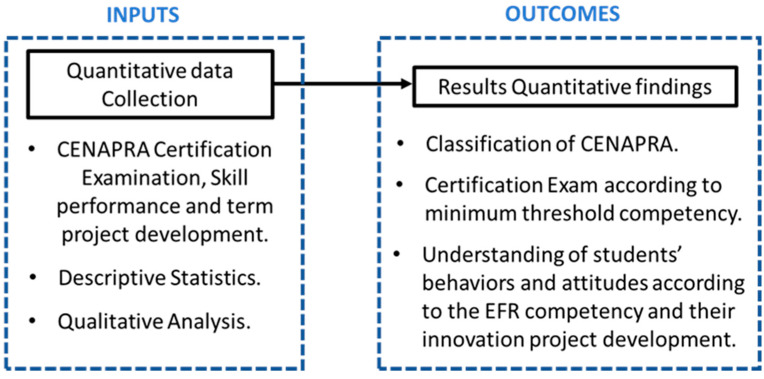
Inputs and outcomes of the sequential quantitative method for the evaluation of the EFR competency.

A quantitative design was selected to conduct this research, as shown in Figure 1. The quantitative design allowed the researchers to elaborate and expand the conclusions about the evaluation of the EFR competency in all students and the domain level according to the CONAPRA certification; the exam was reported during the quantitative results.

### 2.3. Participants

The participating students are freshmen, sophomores, juniors, and seniors from all undergraduate programs at TEC. The EFR project has shown a significant growth in participation since its inception in September 2015. So far, 966 students have participated. They obtain the CONAPRA certification as first responders once they conclude the i-Week and they approve the certification exam in the corresponding year. Table 1 shows the participant distribution and CONAPRA certifications since the project initiated.

From the total number of student participants (966) shown in Table 1, a sample was obtained to perform the quantitative analysis for this study by using the following equation [30]:

(1) $$S=\frac{\chi^{2}\mathrm{NP}\left(1-P\right)}{d^{2}\left(N-1\right)+\chi^{2}P\left(1-P\right)}$$

where N is the population size, P is the proportion of students that are expected to obtain a positive evaluation (P = 0.5 can be used as well), d is the degree of precision that is reflected in the error size (d = 0.05 is recommended), and $\chi^{2}$ is the chi-squared value for 0.95 degree of confidence for the given degrees of freedom in the evaluation. Therefore, Table 2 shows the evaluation of this equation, considering 7 degrees of freedom in the passing domain levels for the CONAPRA certification exam. The sample size used 8 student groups with 23 to 25 students, approximately. This ended up with a sample of 197 students (between 177 and 213) considering the group sizes available and eliminating outliers.

Moreover, Figure 1 shows a block diagram of the sequence of procedures performed to develop the quantitative method to evaluate the EFR competency in students. The figure shows inputs on the left: data collection, EFR evaluation (certification), quantitative analysis on the statistics. In the outcomes, the right side presents the classification level of the CONAPRA certification, as well as the performance of the students in the behaviors and attitudes according to the EFR competency and innovation project development.

The sample of 197 students was used in the quantitative study, after 4 outliers were removed due to their lack of evidence and they were withdrawn from the workshop.

**Figure 2:**
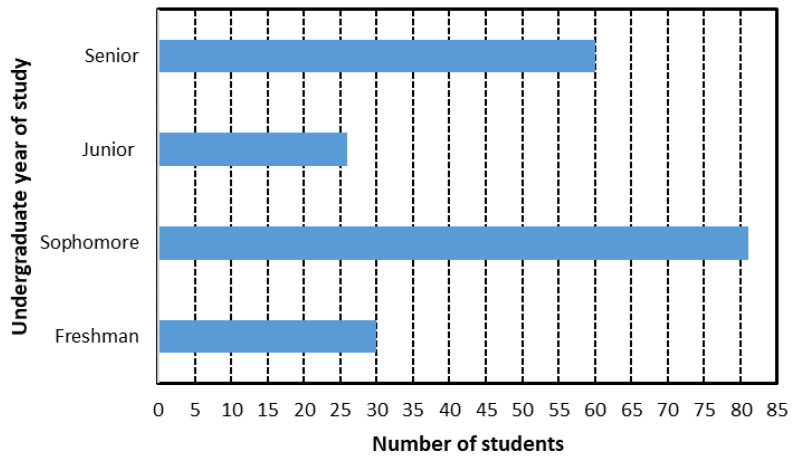
Student sample from 1st to 4th year of college study.

**Figure 3:**
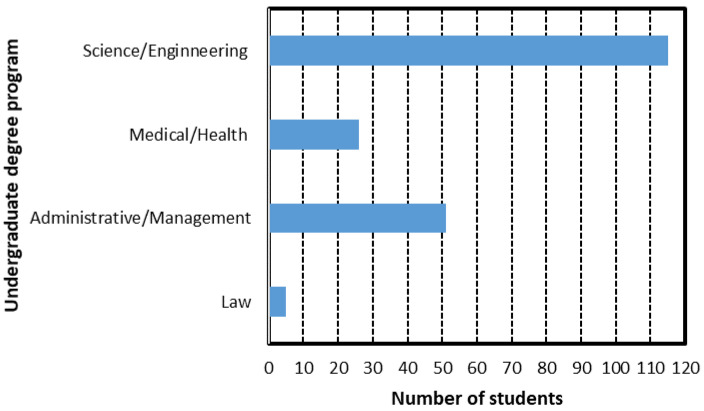
Distribution of the student sample by degree program: Science and Engineering, Medical/Health, Administrative/Management, and Law studies.

Figure 2 and Figure 3 show bar diagrams with the distribution of students from the sample. Figure 2 illustrates the distribution by semester of study where the number of 3rd semester students dominate, with more than 60. In terms of degree program groups, Figure 3 illustrates that science and engineering students dominate the sample, with more than 110 students.

### 2.4. Quantitative Study Method

*Data collection*: This research study used the CONAPRA certification examination (Secretaría de Salud, México, Manual de Formación) [31], and the results of the developed project to measure participants’ competence as “*emergency first responders*”. The exam consisted of 20 multiple choice questions that address different cases from the following conceptual contents reviewed during the first responder workshop:

1. Evaluation of the accident scene;
2. Evaluation of the victims;
3. Choking maneuver;
4. Cardio-pulmonary reanimation (CPR);
5. Wounds, burns, fractures, and convulsions;
6. Bandages and victim dragging.

The developed project consisted of a capstone activity that consolidates the student collaborative work during the i-Week; specifically, the design and integration tool or proposal that contributes to supporting first responder activities. Therefore, this design or proposal was to be oriented toward social organizations, educational institutions, enterprises, offices, commercial outfits, sports clubs, and others. The proposal could include the implementation of a procedure, technique, or method to support education and training about first responder skills and participation in the community. In this case, teams of 5 to 6 persons developed the procedure, specifications, and necessary information to create the tool or app product. As a result, an evaluation rubric was created, and some of the specifications for achieving a reasonable proposal, in order to obtain a practical tool, are shown below:

1. The tool must be proposed to an educational institution, service provider business, company or even the healthcare state administration.
2. The main idea is to advance in creativity and innovation by integrating the knowledge acquired to propose a very useful, informative, servicing, campaigning, interactive tool or app, which could educate, train, or upskill a specified population sector pertaining the first responder in medical emergencies.
3. The tool for emergency response should be selected to support users and workers in preventing and servicing medical emergencies.
4. A presentation about the final activity may include a video or an application, and all the team members must be available during the presentation.

The evaluation rubric was used to evaluate the proposal and it includes items such as:

- The designed tool fulfills the goal of informing and educating about medical emergencies;
- The selected institution and organization exists, and they are willing to use the application;
- The selected project is practical, easy to use, and innovative.

In addition, students answered the CONAPRA certification exam at the end of the week and every team developed a presentation about the capstone activity which was evaluated according to the rubric generated for the final proposal.

*Data Analysis*: The data collected with the CONAPRA certification exam (from a sample of N = 197 students) and project presentation results were analyzed using descriptive statistics to classify participants in domain levels. The average grade between the CONAPRA certification exam and the project presentation was obtained to determine the corresponding competence’s domain level. Using the examination grade (CE) and the project-proposal grade (PG), a final grade (FG) was calculated for each student. Using FG as an indicator, three certified domain levels were defined:

a. Domain level 3, FG=Average(CE,PG) ≥90
b. Domain level 2, FG=Average(CE,PG) 80≤FG<90
c. Domain level 1, FG=Average(CE,PG) 70≤FG<80
d. Non-certified, FG=Average(CE,PG)<70

The three certified homogenous subgroups based on their domain levels defined above were used to collect data. Statistical analysis was used to identify the students’ self-efficacy in the EFR competency; following those indicators, the data collected from CONAPRA certification exam and project proposal results were used in determining the students’ domain level.

*Quality Considerations*: The adapted version of the CONAPRA exam was validated using a previously developed examination used by Secretaria de Salud (Secretaría de Salud, México, Manual de Formación) [31], incorporating 14 professors from Computer Science, Mechatronics/Electrical Engineering and Social Sciences at TEC [32]. Four different exams were designed and the consolidation from 10 to 20 questions was constructed and used to test the students’ competency. Additionally, the Secretaría de Salud as a state institution has personally requested that all the students have correctly performed the main medical emergency drills (procedural contents) of cardio-pulmonary reanimation (CPR), choking maneuver, and scene evaluation to make sure that they had the necessary competence skills to receive the CONAPRA certification. In addition, conceptual contents, and the practical validation of the drill were also evaluated by asking qualitative experts and CPR instructors at TEC about the clarity and students’ possible interpretation of the evaluation drills and question items. These feedback-guided modifications and rewording of some questions/survey items ensured that the items were understandable for participants and that they were all measuring the same concept through the instrument [33].

## 3. Results

**Figure 4:**
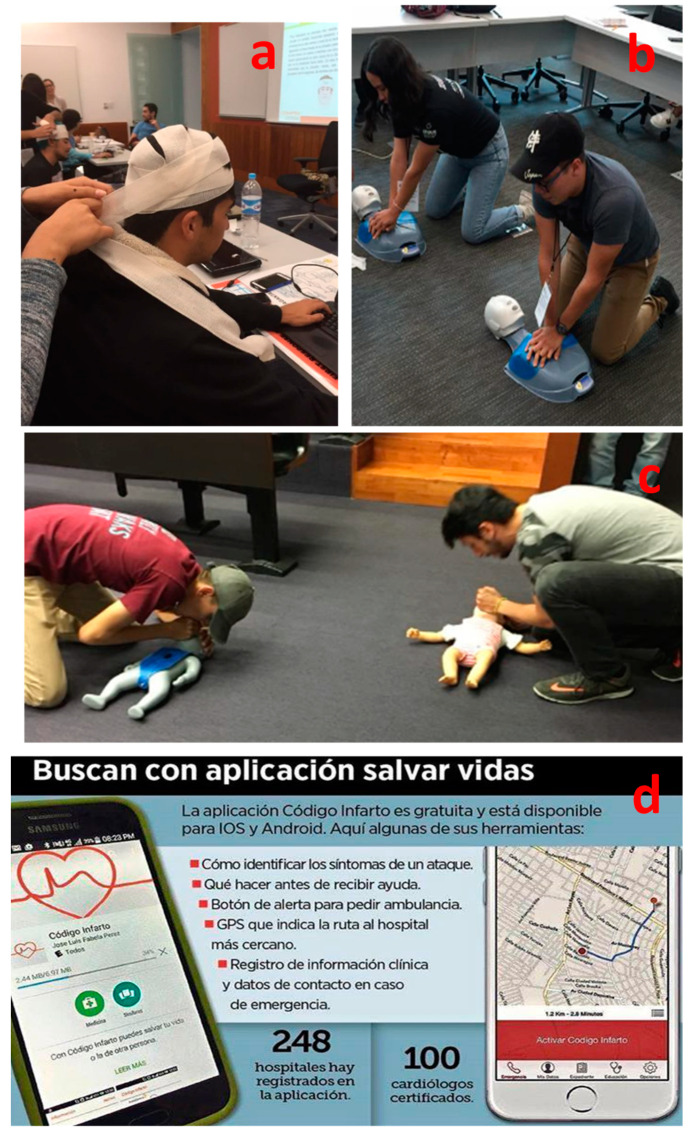
Representative photographs of the theoretical training and practical activities performed in plenary and training sessions during the EFR i-Week at TEC, from 2015 to 2019. (**a**) Photos of practice with bandages during in September 2016. (**b**) CPR training practice and evaluation during the EFR in 2018. (**c**) Same as (**b**), but for babies. (**d**) One of the leading applications generated during the EFR-2015: “An application to save lives using an app for first responders”.

As it has been described above, the i-Week is a EFR workshop with an evaluation that enables participants to obtain the CONAPRA certification in three different levels. Figure 4 shows photos of some EFR i-Week activities from 2015 to 2019.

Figure 4a shows a bandages practice; Figure 4b,c describe a CPR training practice and evaluation, and Figure 4d shows a leading application generated during the EFR to save lives using an app for first responders named “codigo Infarto”. This project and developed app were made using both the IOS and the Android platforms. The app shows information on how to identify the symptoms of an attack and what to do before the health professionals arrive. It also includes an alert button to request an ambulance and a GPS that indicates the route to the nearest hospital, and contains a record of clinical information such as emergency contact details.

### Quantitative Results

The EFR competency development and desired outcomes for the students included:

- The certification from CONAPRA (50%).
  a. A successful observation and evaluation by instructors of a practice realization of the drills required: accident scene evaluation, shocking maneuver, CPR procedure and good development of bandages, lifting carries.
  b. A successful passing of the certification exam from CONAPRA.
- The development and presentation of the capstone project proposal to support the EFR initiative in communities (50%).

The final “competency evaluation” consisted of 50% CONAPRA certification and 50% the capstone project. 

**Figure 5:**
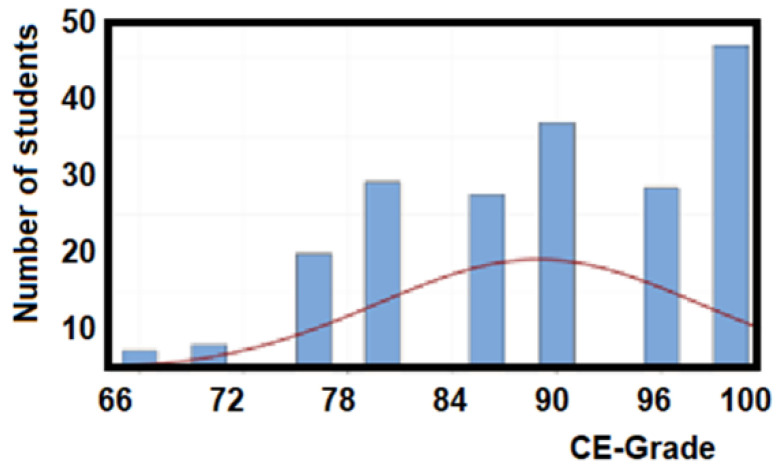
Statistics of certification grade (CE) for the EFR competency.

Figure 5 illustrates that the sample population obtains a mean of 88.9/100 in the certification grade with a standard deviation of 9.19. Additionally, a 95% confidence interval for the mean is obtained from 87.6 and 90.2.

**Figure 6:**
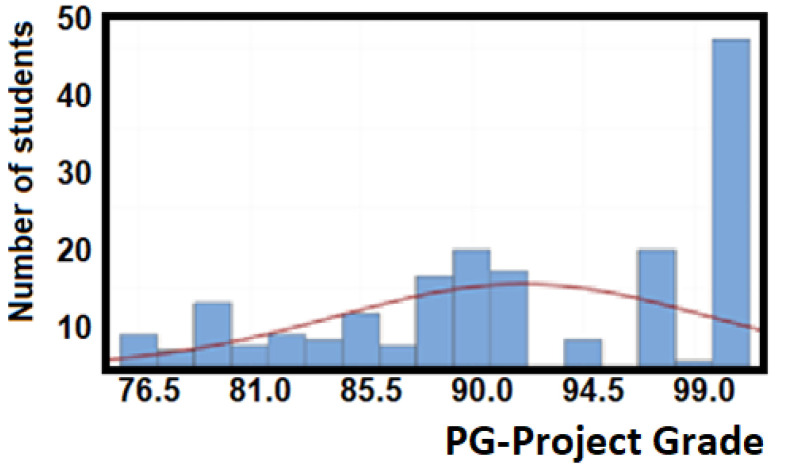
Statistics of project grade (PG) for the EFR competency.

Likewise, Figure 6 illustrates that the sample population obtains a mean of 92/100 in the capstone project grade with a standard deviation of 7.56. The data show that a 95% confidence interval for the mean is obtained from 90.98 and 93.11. In both cases, most students are in the competency domain levels 2 and 3.

**Figure 7:**
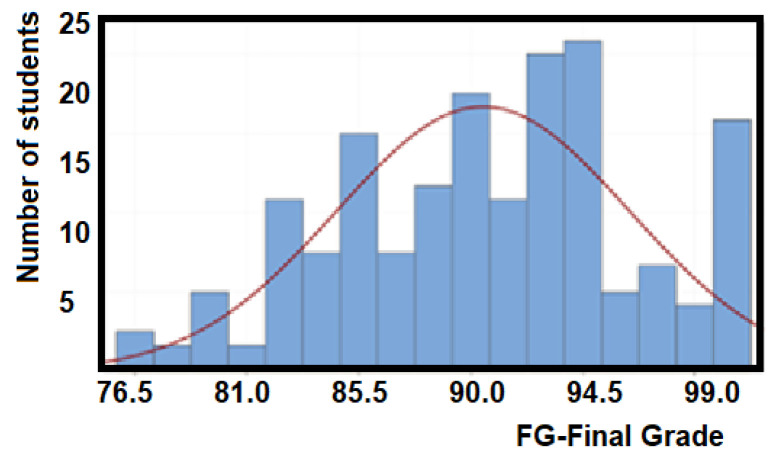
Statistics of the final grade (FG), which is added the contribution of the examination grade (CE), and the project grade (PG) for the EFR competency.

Figure 7 illustrates the combination of both results with a mean value of 90.49/100 in the final-grade and a standard deviation of 5.88, which indicates a higher performance with less dispersion. Additionally, a 95% confidence interval:

5. For the mean is obtained from 89.66 to 91.32, and
6. For the median is obtained from 90.00 to 92.5.

**Figure 8:**
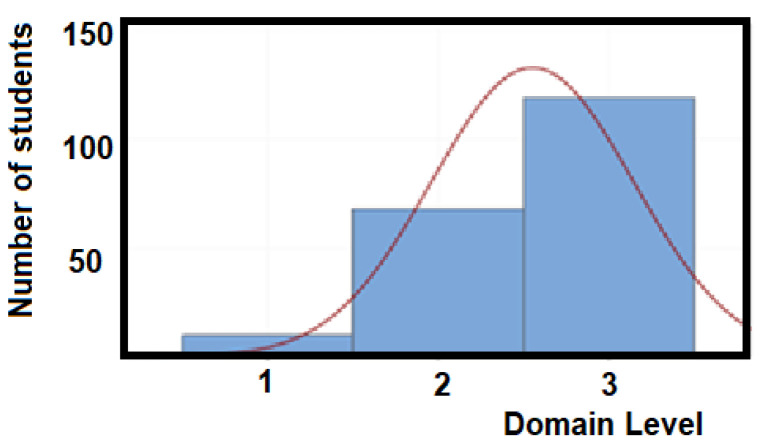
Graph of the domain level for the EFR competency. Mean = 2.55, standard deviation = 0.59 and 95% confidence interval for mean (2.47:2.63).

This suggests that most students are very close to the highest EFR competency domain level (level 3), as illustrated by the statistics shown in Figure 8.

Following theoretical selection guidelines [7], the participants were associated, both from each of their undergraduate years of study: freshman (1), sophomore (2), junior (3), and senior (4); and from the four fields of study categories (1 to 4) as follows:

1. Science and Engineering (Science, Technology, Engineering and Math areas).
2. Medical and Health Sciences.
3. Administrative and Management Sciences.
4. Law.

**Figure 9:**
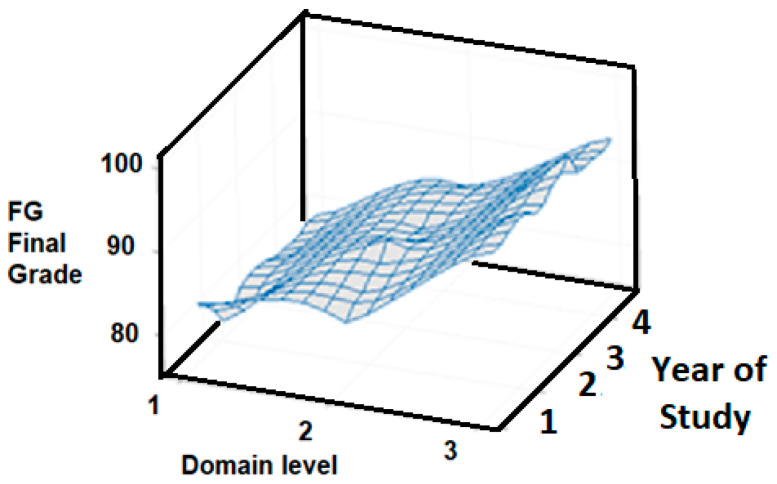
3D Surface graph of final grade vs. semester (with the domain level as the *x* axis variable) for the emergency first response competency.

**Figure 10:**
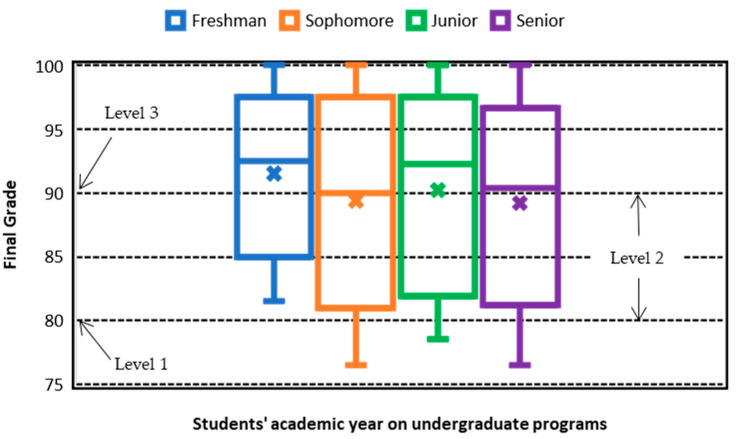
Box and whisker plot on the final grade vs. undergraduate year for the EFR competency.

These groups should complement and test the emerging theory of their field of study, and their university level does not necessarily affect their performance evaluation in the EFR competency evaluation. The final EFR competency evaluation is obtained by combining the results from Figure 9 and Figure 10 for the sample of 197 students. Figure 9 shows a 3D plot for the final grade versus undergraduate year (*y*-axis) and domain level (*x*-axis). The graph shows that the variation in the final grade with respect to the undergraduate year of study (from 1 to 4) is essentially negligible for the EFR competency.

Figure 10 presents the box and whisker plot of the final grade, with respect to the independent variable “undergraduate year”. The results indicate that most of the final grades are roughly between 75 and 100, independent of the students’ semester of college study. However, there is a little less variation in the freshman grades. As a result, the information of the grades associated with the quartile 1, 2, 3, and 4 and the median remain approximately the same for all undergraduate years. Therefore, the essential observation is that there is no additional advantage in the EFR competency in the advanced undergraduate years, to obtain the maximum level of certification (level 3).

**Figure 11:**
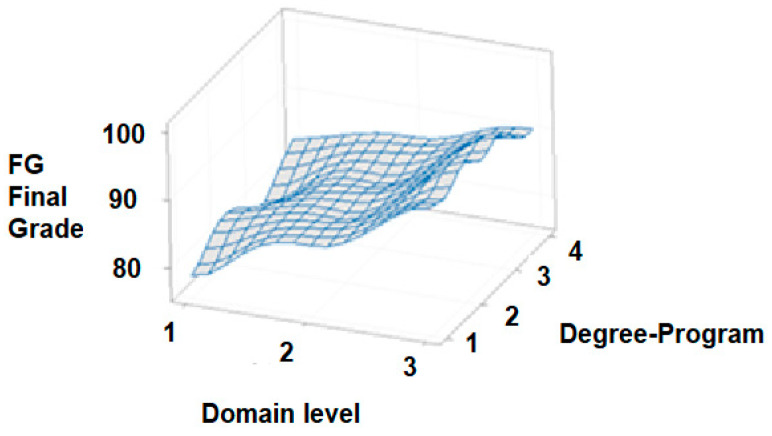
3D Surface graph of final grade vs. degree program group (with the domain level as the *x* axis variable) for the EFR competency. The degree program group is described as follow: (1 = Science/Engineering, 2 = Medical Sciences, 3 = Administration/Management and 4 = Law).

Continuing with the revision of the independent variable of students’ degree pro-gram, Figure 11 shows another 3D plot for the final grade versus degree program (*y*-axis) and domain level (*x*-axis). As illustrated before, this graph shows that the variation of the final grade with respect to the degree-program group (1, 2, 3, and 4) is essentially negligible for the EFR competency.

**Figure 12:**
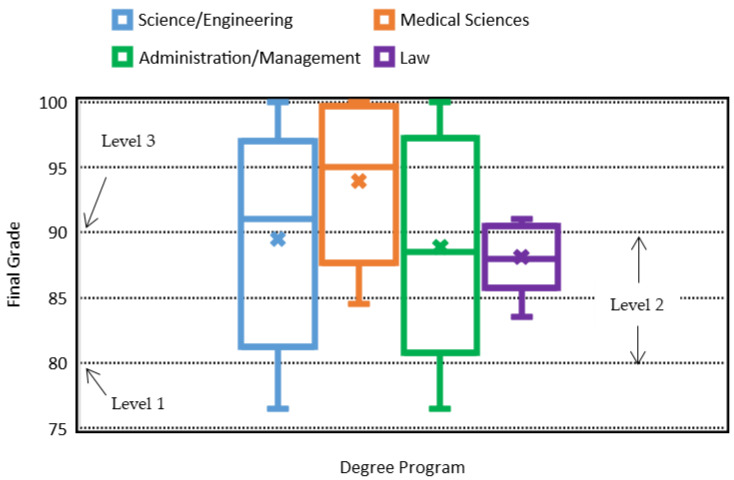
Box and whisker plot on the final grade vs. degree-program group (1 = Science/Engineering, 2 = Medical Sciences, 3 = Administration/Management and 4 = Law) for the EFR competency.

Moreover, Figure 12 shows information from the box and whisker plot where the final grade versus degree of undergraduate program is correlated. Specifically, it is observed that undergraduate students in the areas of science and engineering, as well as administration and management, show approximately the same behavior, where there is greater variability between quartiles and final grades. However, this variability between quartiles has been mitigated in the programs of medical areas; that little variability of the quartiles is even more present in the law areas. In the various areas of undergraduate programs, final grades were presented with the highest scores of 100, except for law programs. On the other hand, the lowest final grades were not presented in the law program but in the areas of science and engineering, and administration and management. As a group, it can be observed that in general terms, students from medical areas present the best final grades on an average basis. This is not necessarily indicative that the healthcare areas in the undergraduate programs have a predetermined advantage of obtaining the maximum level of certification (3), since in the other disciplines, high final grades were also observed to acquire the maximum level. However, it is possible that the average final grades were a little higher in medical areas due to the close connection with this course; in addition, this i-Week generates greater interest and commitment in the medical students compared to the students of the other programs. Likewise, it can be indicated that the final grades of the undergraduate law program are mostly below the minimum qualification to acquire level 3 of the certification; this means, in this discipline, most of the students did not acquire the maximum level of certification.

**Figure 13:**
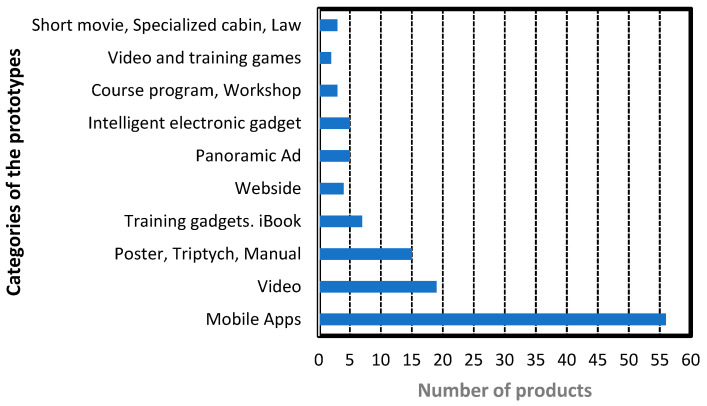
Typical categories of the product prototype generated during the EFR i-Week from 2015 to 2019.

Regarding the students’ delivered products, personnel from SSNL and CONAPRA were part of the evaluation committee in the final oral presentations. Figure 13 illustrates a sample of categories for different product prototypes developed by the students from 2015 to 2019 in the EFR i-Week. SSNL and CONAPRA were very optimistic about the use of the generated products and applications. The multidisciplinary and collaborative [34,35,36,37,38] approach to the EFR project was so successful that the i-Week, now called “Week-TEC”, will continue during the years to come.

Other specific accessory development was observed with more advanced students, who initiated designs of first responding devices such as shields (clear mouth barriers for artificial ventilation systems) or rescue masks for CPR procedures [39]. Additionally, the interaction among students from mechatronics, electrical engineering, computer science and information technologies [40] in the development of technology-driven applications [41], and the participation of companies [42] provides a very fertile multidisciplinary environment (similar to engineering clinics) to promote innovation and new business opportunities. Figure 13 shows the different prototype categories of products developed by students in which mobile applications predominate as the prototype product.

Likewise, the progress observed in i-Week derived from the products made by the students not only shows a greater engagement in the students to their learning in the course, but also shows that the great differentiator of this course was the addition of these product-producing activities carried out by the students on top of the fact that the course was available for all undergraduate majors. As a result, these findings are so far unpublished and relevant to the available literature regarding EFR study courses, and they can help to reach a dynamic change in higher education.

## 4. Discussion

The population of college students with a wide variety of major fields of study at TEC in this study reported a high domain level in the EFR competency after completing the workshop and project development in the i-Week at TEC. Additionally, considering that the students ranged from freshman to seniors, they consolidated their ability to perform practical skills and innovation regarding providing support to the EFR concept in the community. In addition, students’ confidence in completing all the activities, presentations and proposals increased enormously, according to their results of the level of certification obtained.

Most of the participants in this study were very motivated about the citizenship element and participation of different sectors and were eager to apply their proposals to different institutions in the community; according to the evidenced final grades, the success of their products that were obtained under the workshop, and according to with the enrollment numbers register for the course, regardless of the area of discipline of the undergraduate programs of the students.

Most students achieved the highest domain level of 3 in the EFR competency, and in terms of CONAPRA and the proposal evaluation, the sample group had a mean of 2.55 and a median of 3. From the standpoint of the health department certification [31,43], CONAPRA elaborates the registered certificates and sends them to TEC authorities in a period of about 1 year. The opportunity areas for the improvement of some details in the workshop were addressed by faculty and health officials, year by year, from 2016 onwards. This has allowed a much better performance from the low end, that is, students with domain level 1. This new and more realistic perception of their EFR competency was related to a noticeable change in participants’ behavior and attitudes when performing the practical skills, such as CPR, and developing the proposed projects for different institutions. By adjusting their approach to the product prototype or proposal, participants were able to develop even further their EFR competence, up to the point that some of them could reach the highest domain level (level 3), during the project presentation to their classmates.

### Limitations

This research should be viewed considering the application of a new instructional model of general education for colleges and universities, implementing competency-based degree programs with robust interaction from industry, institutions and government organizations in student education. The required domain level of different competencies can be achieved by students during their 4 years of college studies, and this EFR could be integrated into the students’ life-plan of activities. TEC initiated degree-plans-TEC-21 during the fall semester of 2019, and this new approach in college education involves not only subject percentage grade evaluation, but also competence domain level evaluation. This also includes the industrial partner (in this case study, the State’s Health Department, SSNL, participated), which reinforces the commitment to integrating the community’s instructional teams so that they participate in the whole educational process. This strategy of including a good industrial, organizational, or government partner plays a key role in the successful completion of the competence-based education that is presented in this general education example. The experience with SSNL has been so successful that the continuation of the project has been authorized for many years to come. This partnership between TEC and SSNL has generated more than 1000 new first responders since 2015. Additionally, the performance trend results from TEC students are representative of a particular student population at TEC; thus, it is recommended that the appropriate adjustments are made in order to implement this course according to the resources and needs of each academic institution. As a result, it should not be the intention to pursue a generalization of behaviors and attitudes to all college students in Mexico and worldwide, but certainly this study with successful results of this workshop has shown that these EFR courses are necessary for students with different majors in order to achieve the benefit of risk prevention at work, in our mobility and at home.

## 5. Conclusions

This research contributes to the general education competencies in order to encourage the students to participate in citizenship and their community by building up their EFR competency in their day-to-day life activities. This research initiated a reflection regarding questions R.Q.1, R.Q.2, R.Q.3, and R.Q.4. The discussion and answers were provided in the paper, and a summary is given below.

R.Q.1 What relationship exists between the CONAPRA certification exam performance and the EFR competency acquisition by undergraduate students?

Quick Review Answer to R.Q.1. From a population of 966 students and a sample population of 197, the mean grade in the CE exam was 88.9/100 with a standard deviation of 9.19. Additionally, the median was 90, indicating that most of the students are between skill levels 2 and 3 of the EFR competency, taking into consideration that the practice (procedural contents) drills were performed and verified by the state health department.

R.Q. 2 What are student behaviors and attitudes towards the generation of contributions and innovations to the first responder community in Mexico?

Quick Review Answer to R.Q.2. This answer is focused on the college student population at TEC, which may not represent the overall college students in Mexico. Considering the student behaviors and attitudes towards innovation and making contributions, a sample of categories for different product prototypes developed by the students from 2015 to 2019 in the EFR i-Week shows a very positive outcome for the EFR competency. The multidisciplinary and collaborative nature of the EFR project proposals was so successful that, over the years, many contributions went on to follow them up with the best applications, prototypes, and proposals. The sample contributions are mobile apps, videos, posters/triptychs, training gadgets/iBooks, websites, panoramic-ad, dedicated hardware devices, course programs, short movies, video games, laws, and others. Additionally, the participation of faculty members from different departments such as mechatronics and electrical engineering, computer science, and information technologies promoted the development of technology-driven applications, with the participation of industrial partners. In a nutshell, from the point of view of providing a research study for an EFR workshop for undergraduate students for all majors, as well as by including in this workshop the activity of contributing with a product used for EFR, this approach shows a great differentiation with respect to the studies available in the literature.

R.Q. 3 What differentiation exists between domain levels, 1, 2, or 3, when students are certified by the final grade that includes the CONAPRA exam to obtain the EFR competency, and their major discipline of study (science/engineering, administrative/management studies, medical professions, or law)?

Quick Review Answer to R.Q. 3. This study obtained an analysis of the quartiles, and median of the final grade (FG) and the major discipline (1 = science/engineering, 2 = administrative/management, 3 = medical or 4 = law). In science/engineering disciplines, as in administration/ management areas, there was greater variability in the final grades; on the other hand, in the discipline of law, the variability of final grades was lower. Likewise, in the areas of law, the students did not obtain a grade of 100 as they did in the other areas, but nor did law students show the lowest grades; however, it was shown that in the area of law, very few students obtained the highest level of certification. Although the median of the final grade in the area of medicine is slightly higher than the averages of the other areas, this behavior is certainly not a condition for obtaining the highest certification level for students of other disciplines. As a result, we believe that student product development for this course goes a long way in linking all undergraduate programs to the subject of EFR; consequently, this activity has shown an increase in the engagement of students to obtain higher performance scores.

R.Q. 4 What differentiation exists between domain levels, 1, 2 or 3, when students are certified by the final grade that includes the CONAPRA exam to obtain the EFR competency, and their year of study (from freshmen, sophomores, juniors, and seniors)?

Quick Review Answer to R.Q. 4. The results of the quartiles and the median are approximately the same regardless of the level of the year of college study, with final grades between approximately 75 and 100. However, there is slightly less variability in the final grade in the freshman year. In summary, regardless of the study year of the students, the results show that students can obtain the maximum level of certification; therefore, this shows that there is no advantage between the years of the undergraduate program. Likewise, the variation in the final grade shows, once again, that most students obtained domain levels between of 2 and 3 in the EFR competency after successfully completing the EFR course, regardless of their year of study.

Despite some negative behaviors and attitudes related to very few college students who show lower EFR competency, some students are very likely to participate again in the EFR project, regardless of facing negative experiences because of absence marks or failure to succeed in the drill evaluations, and/or the certification exam. Faculty members should try to take advantage of this persistence shown by those students, and try to engage them in assessment and consulting activities with other newcomer students. Moreover, students with high levels of performance (domain level 3) in the EFR competency, or even some medical sciences students, are likely to respond positively to a professor’s constructive feedback about their EFR drill deficiencies, as their performance self-efficacy enables them to enjoy working on assessing other classmates in related activities, but also to feel capable of performing well in them.

### Future Work

Future research should be conducted to determine the evaluation and validation of other disciplinary and general education competences in engineering and all other college degree programs. Additionally, the evaluation should incorporate other instruments and evidence to include additional attitudinal contents. Finally, with the new reality that exists with the presence of COVID-19, our next EFR courses will have to consider measurements of the impacts of pandemic experienced by students.

## Figures and Tables

**Table 1:** Student participation and certifications by CONAPRA from 2015 to 2019.

Year	Students	EFR Professors	Other Professors or Collaborators
2015	182	23	12
2016	198	4	22
2017	175	1	0
2018	209	7	0
2019	202	0	0
Total	966	35	24

**Table 2:** Quick estimation of a minimum sample size for a population of 966 [30].

N	P	d	Χ^2^	Deg-Free	S
966	0.5	0.05	0.0039	1	0.4
966	0.5	0.05	0.1026	2	10.2
966	0.5	0.05	0.3518	3	34.0
966	0.5	0.05	0.7107	4	66.3
966	0.5	0.05	1.1455	5	102.5
966	0.5	0.05	1.6354	6	140.0
966	0.5	0.05	2.1673	7	177.2
966	0.5	0.05	2.7326	8	213.2

**Figure A1:**
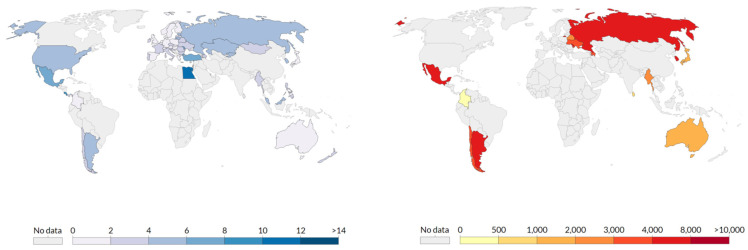
Data for the worldwide population. (**a**) Fatal occupational injury rates, 2017; the frequency rates of fatal occupational injures measure as the number of cases of fatal injury per 100,000 employees. (**b**) Non-fatal occupational injury rates, 2017; the frequency of non-fatal occupational injuries, given as the number of cases per 100,000 employees. Information provided by United Nations Statistics Division (2019) in “Fatal occupational injury rates” for (**a**), and “Non-fatal occupational injury rates” for (**b**), maps published online at OurWorldInData [44].

**Figure A2:**
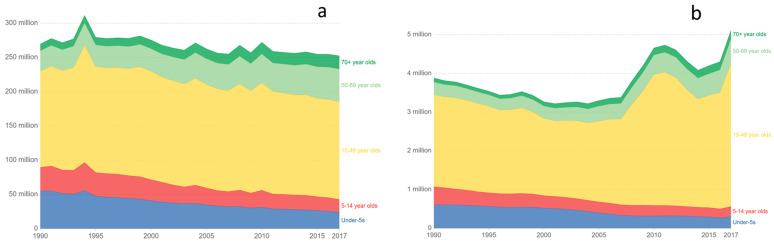
Burden of disease from injuries by age from 1990 to 2017 (**a**) Data for the worldwide population. (**b**) Data for the Mexican population. Disease burden is measured in disability-adjusted life years (DALYs). DALYs are used to measure the total impact of disease, both from years of life lost and years lived with a disability. One DALY equals one lost year of healthy life. Information provided by Institute for Health Metrics and Evaluation [45].

**Figure A3:**
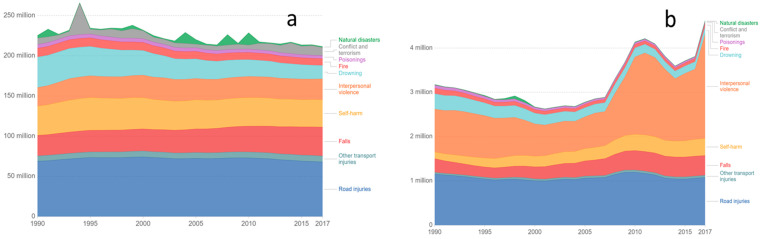
Burden of disease on the classification of injuries from 1990 to 2017. (**a**) Data for the worldwide population. (**b**) Data for the Mexican population. Disease burden is measured in disability-adjusted life years (DALYs). DALYs are used to measure total of disease—both from years of life lost and years lived with a disability. One DALY equals one lost year of healthy life. Information provided by Institute for Health Metrics and Evaluation [45].

**Figure A4:**
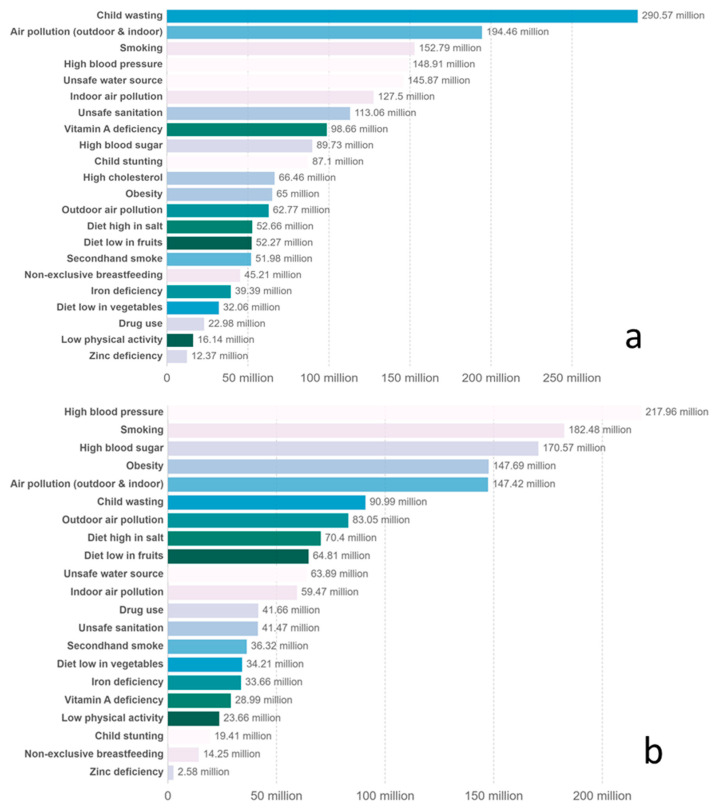
Disease burden by risk factor. (**a**) Data for the worldwide population in 1991. (**b**) Data for the worldwide population in 2017. The disease burden is measured as disability-adjusted life years (DALYs). One DALY is the equivalent of losing one year of good health because of either disability or premature mortality. One DALY represents one lost year of healthy life. Information provided by Institute for Health Metrics and Evaluation [45].

**Figure A5:**
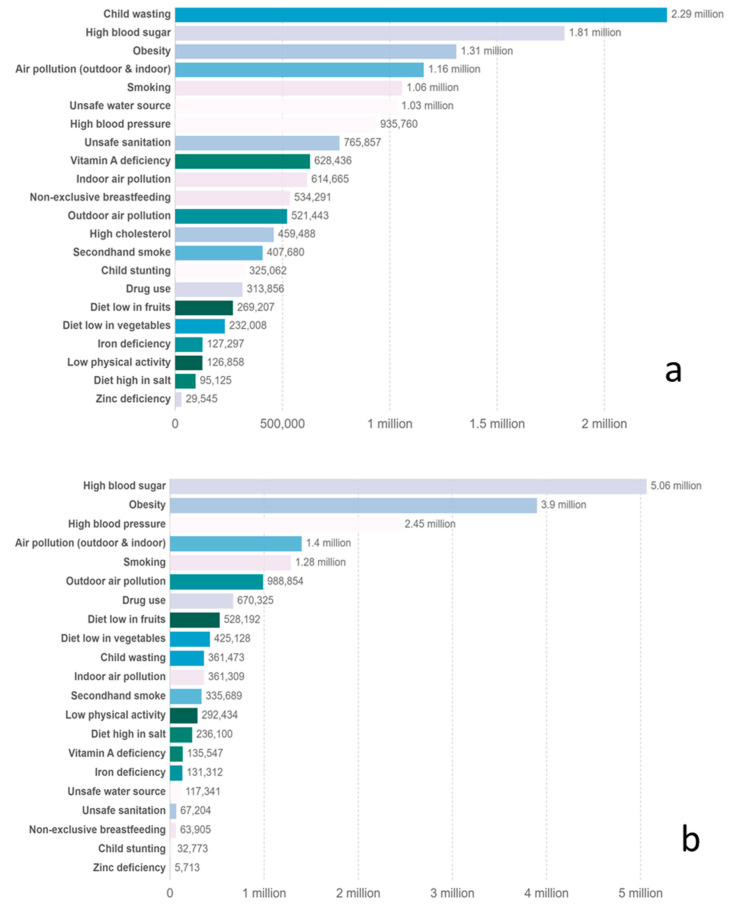
Disease burden by risk factor. (**a**) Data for the Mexican population in 1991. (**b**) Data for the Mexican population in 2017. The disease burden is measured as disability-adjusted life years (DALYs). One DALY is the equivalent of losing one year of good health because of either disability or premature mortality. One DALY represents one lost year of healthy life. Information provided by Institute for Health Metrics and Evaluation [45].

**Figure A6:**
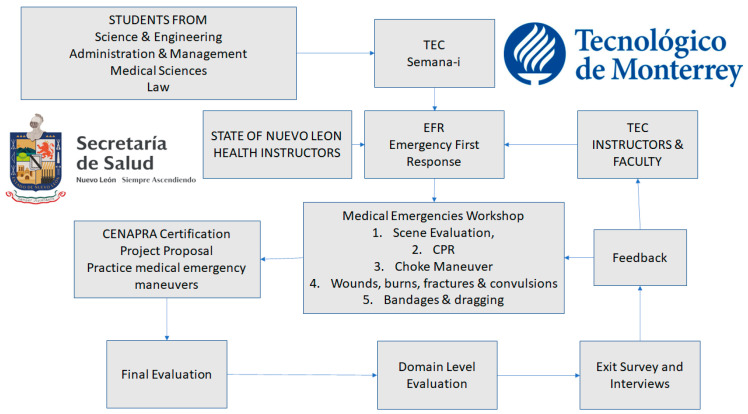
Process interactions in the EFR study for college students at TEC, Secretaría de Salud (Nuevo León State, México) and the “emergency first response” competency evaluation of the student population.

**Figure A7:**
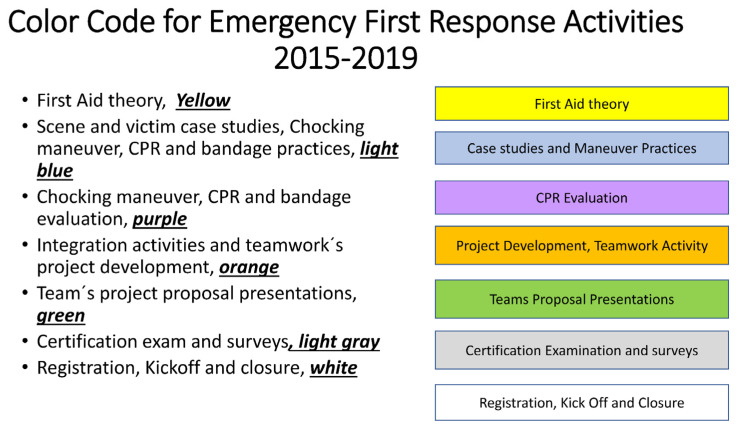

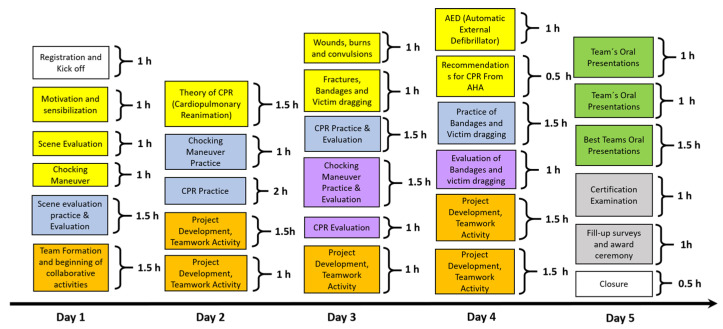
Color code and 5-day blocks of scheduled activities for the EFR workshop.

## Appendix A. Work Occupational Safety Worldwide and in Mexico

Since the need to manage people’s “health and well-being” according to objective 3 of the SDG has been established in the introduction section, people’s lifestyle or habits need to be investigated. For instance, if it is contemplated that a third of the day people are resting, then the active lives of people are developed mainly in their workplace, where transportation needs are required. Therefore, to offer better health and safety to people, it is essential to know the rate of deaths and incidents due to injuries in the workplace. Figure A1 shows worldwide data from 2017; (a) the fatal occupational injury rates, and (b) the non-fatal occupational injury rate. The world map in Figure A1a shows that there are several countries where no information is available; however, the countries with the highest incidence of fatality associated with the workplace are Egypt, Turkey, Mexico, Kazakhstan, Argentina, Myanmar, the United States and Russia, which establishes that these countries have an incidence rate of approximately between 4 and 12 people per 100,000 employees, according to the intensity of the blue color.

The world map in Figure A1b also shows that no data are available for several countries; however, the countries with the highest incidence of injuries associated with the workplace are Mexico, Argentina, Russia, South Korea, Myanmar, Japan, Australia, Chile, Ukraine, Moldova, Belarus, and Azerbaijan, which states that these countries have an approximate incidence rate of between 1000 and 8000 people for every 100,000 employees, according to the intensity of the orange-red color.

Figure A2a,b it is shown the main disease burdens due to injuries by age in people in the years from 1990 to 2017, worldwide and in Mexico, respectively. In Figure A2a,b, it is observed that the data show a downward trend in the burden of illness by lessons in people under 14 years of age. For the age range of 15 to 49 years, there is an approximately unchanged trend for the data at the world level, but in Mexico it is on the rise, which is worrying.

In the case of the age group over 50 years of age, there are no significant changes in the period range of time reported in Figure A2a,b. Therefore, it can be concluded that the greatest number of accidents in people occurs in their roughly productive working age, which is about 15 to 50 years old.

The information reported in Figure A2a,b can also be analyzed with respect to the main causes of injury burden on people. For example, Figure A3a,b, it is shown the main causes of injuries worldwide and in Mexico, respectively; the main injury causes are associated with roads accidents, falls, self-harm, interpersonal violence, and drowning. In the case of the worldwide data, road injures are the leading cause; in contrast, the leading cause of accidents in Mexico is interpersonal violence.

Therefore, given that there is a significant burden of injuries, both in world data and in Mexico, linked to activities related to the workplace, it can be observed that people should have knowledge in EFR; in addition, it is significantly relevant for the case of Mexico to take the responsibility to develop social programs that teach people social communication, which will reduce the occurrence of interpersonal violence.

In addition, Figure A4a,b it is shown a comparison of the burden of disease by risk factor worldwide for 1991 and 2017, respectively. For world data, it is observed that in recent years there has been an increase in risk factors in the burden of diseases related to high blood pressure, high blood sugar, and obesity. This tendency is in line with the Mexico data observed in Figure A5a,b. Therefore, the burden of disease by risk factor that has been presented in recent years shows a poor level of health in people, where occupational accidents can increase if this trend continues.

## Appendix B. Inter-Institutional Administrative Structure and EFR i-Week Workshop Schedule

The project was developed in collaboration with the following entities:

1. EIC (School of Engineering and Sciences at TEC);
2. EGADE (Graduate School of Management and Business Administration at TEC);
3. Health Department at the State of Nuevo León (SSNL);
4. National Center for Accident Prevention in México (CONAPRA);
5. Ternium in Monterrey (TERNIUM);
6. Solvay in Monterrey (SOLVAY).

Figure A6 illustrates the process interactions required to perform this study.

Moreover, Figure A7 shows the color code and distribution of the activities during the workshop. The figure illustrates the amount of time dedicated to main activities as follows:

IV. First Aid theory (yellow, 8 h).
V. Scene of victim case studies, chocking of maneuvers, CPR and bandage practices (light blue, 7.5 h).
VI. Evaluations of chocking of maneuvers, CPR, and bandage (purple, 3.5 h).
VII. Teamwork activities (orange, 8 h).
VIII. Proposal presentation of projects by teams (green, 3.5 h).
IX. Certification exam, and surveys (gray, 2 h).
X. Kickoff and closure (white, 1.5 h).

Other non-scheduled activities not shown in the diagram are: lunch 6 h, transportation to workshop site 3 h, and breaks 1 h. Therefore, the total number of hours during the week are 30 h of full project dedication and 10 h of other non-scheduled activities.
